# Effects of Cardiomyocyte-Specific Deletion of STAT3–A Murine Model of Heart Failure With Preserved Ejection Fraction

**DOI:** 10.3389/fcvm.2020.613123

**Published:** 2020-12-07

**Authors:** Weilin Zhao, Yanjia Chen, Wenbo Yang, Yanxin Han, Zhiyan Wang, Fanyi Huang, Zeping Qiu, Ke Yang, Wei Jin

**Affiliations:** ^1^Department of Vascular & Cardiology, Ruijin Hospital, Shanghai Jiao Tong University School of Medicine, Shanghai, China; ^2^Institute of Cardiovascular Diseases, Shanghai Jiao Tong University School of Medicine, Shanghai, China

**Keywords:** HFPEF, stat3, fibrosis, cardiac hypertrophy, passive stiffness

## Abstract

**Aims:** There is a high incidence of heart failure with preserved ejection fraction (HFpEF), but the options of treatment are limited. A new animal model of HFpEF is urgently needed for in-depth research on HFpEF. Signal transducer and activator of transcription 3 (STAT3) may affect the passive stiffness of myocardium, which determines cardiac diastolic function. We hypothesized that cardiomyocyte-specific deletion of STAT3 increases cardiac passive stiffness, which results the murine features of HFpEF.

**Methods and Results:** Cardiomyocyte-specific deletion of STAT3 (STAT3cKO) mice was generated by the Cre/FLOXp method. The STAT3cKO mice showed heavier cardiac fibrosis and cardiac hypertrophy comparing with wild-type (WT) mice. Furthermore, STAT3cKO mice showed increased serum brain natriuretic peptide (BNP) level, and growth stimulation expressed gene 2 (ST2) level. Other indicators reflecting cardiac passive stiffness and diastolic function, including end diastolic pressure volume relation, MV A value, MV E value, E/A and E/E' had different fold changes. All these changes were accompanied by decreasing levels of protein kinase G (PKG). Bioinformatic analysis of STAT3cKO mice hearts suggested cGMP-PKG signaling pathway might participate in the pathogenesis of HFpEF by means of adjusting different biological functions.

**Conclusions:** Cardiomyocyte-specific deletion of STAT3 results in a murine HFpEF model which imitates the clinical characteristics partly by affecting cardiac PKG levels. Better understanding of the factors influencing HFpEF may finally provided innovative therapies.

## Introduction

Heart failure (HF) is a complex clinical syndrome caused by various etiologies and can be classified as preserved, mild-range and reduced ejection fraction (EF) (1). According to reports, heart failure with preserved ejection fraction (HFpEF) accounts for more than 50% in all HF patients, and there is no doubt that HFpEF will become the commonest type of HF around the world in the future (2–4). HFpEF is a complex syndrome with high morbidity and mortality. Despite many efforts, so far, there are no evidence-based therapies (5, 6).

In the past few decades, plenty of murine models were developed to simulate diverse pathological mechanisms administering to HFpEF. The most common HFpEF models focus on investigating classic risk factors including hypertension, obesity, diabetes mellitus, and aging (7–11). In addition to these limitations, these classic animal models are highly heterogeneous and do not meet the commonality seen with any specific HFpEF population. Clinical trials have suggested that the cardiac passive stiffness in HFpEF patients increase obviously (12, 13), which is the main characteristic of HFpEF and has been ignored in other animal models. So there is an urgent need to produce new animal models for further resolving these problems.

Signal transducer and activator of transcription 3 (STAT3) can be activated by various cytokines to exert a variety of biological effects (14, 15). Previous studies showed the loss of STAT3 was prone to fibrosis development and other pathogenesis in heart (16). Significantly, cardiomyocyte-specific deletion of STAT3 in mice induced deep reduction of PKG (17), which involved in interstitial fibrosis and cardiomyocyte hypertrophy (18, 19). In diastole, collagen as the major constituent of extracellular matrix contributes mostly to cardiac passive stiffness (19–21). Thus, we aimed to determine if cardiomyocyte-specific deletion of STAT3 could impair cardiac diastolic function in this model. Our data established that cardiomyocyte-specific deletion of STAT3 in mice would lead to cardiac fibrosis, decreased capillary density, cardiac hypertrophy, and eventually impaired cardiac diastolic function partly by reducing the levels of PKG.

## Methods and Materials

### Animal Models

The experimental protocols of all animals were ratified by the Committee on the Ethics of Animal Experiments of Ruijin Hospital. Cardiomyocyte-specific αCre mice and male STAT3 (flox^+/+^) mice at 4 weeks were purchased from the Jackson Laboratory. All of the experimental mice were kept in the Animal Experiment Center of RuiJin Hospital. Flox/flox mice were mated with the tamoxifen-inducible αCre mice. The 8-week-old flox/flox Cre+ mice subjected to intraperitoneal injection of tamoxifen (T5648, Sigma) at a dose of 50 mg/kg/day for 5 consecutive days. When cardiomyocyte-specific deletion of STAT3 had been performed and mice had grown to 4 months old, we tested all the indicators described below.

### Echocardiography

The echocardiology parameters such as left ventricular eject fraction (LVEF), fractional shortening, interventricular septum thickness at end-systole (IVS; s) and end-diastole (IVS; d), the left ventricular posterior wall thickness at end-systole (LVPW; s) and end-diastole (LVPW; d), trans-mitral valve velocity E peak (MV E) and A peak (MV A), E/A and E/E' were all performed by VisualSonics Vero2100 system and achieved from M-mode long-axis views or apical four-chamber views.

### Blood Pressure and Pressure-Volume Measurements

The CODA apparatus and tail-cuff method (Kent Scientific) were used to measure mice systolic blood pressure. All the mice were tested at least eight times. We calculated the mean systolic blood pressure value of repeated measurements. The pressure-volume measurements were achieved by SciSense Advantage Admittance Derived Volume Measurement System and 1.2F catheters (SciSense). Data were captured and analyzed by LabScribe2.

### Western Blot Analysis

The protein samples were achieved from heart tissue. The prepared protein samples with equal amounts were separated by SDS-PAGE and blotted onto polyvinylidene fluoride membranes. The membranes were then incubated with antibodies against STAT3 (1:1000) (Cell signaling technology, cat# 9139; RRID:AB_331757), collagen 1 (1:1000) (Abcam, cat# ab21286; RRID:AB_446161), collagen 3 (1:1000) (Antibodies-Online, cat# ABIN285714; RRID:AB_10789249), fibronection (1:1000) (Abcam, cat# ab2413; RRID:AB_2262874), CD31 (1:1000) (Cell signaling technology, cat# 77699; RRID:AB_2722705), p-phospholamban (1:1000) (Cell signaling technology, cat# 14562; RRID:AB_2798511), p-troponin I (1:1000) (Cell signaling technology, cat# 4004; RRID:AB_2206275), PKG (1:1000) (Cell signaling technology, cat# 3248; RRID:AB_2067450) and GAPDH (1:10000) (MBL International, cat# M171-3; RRID:AB_10597731) at 4°Covernight. The horseradish peroxidase (HRP)-conjugated secondary antibodies were incubated with polyvinylidene fluoride membranes for 1 h at room temperature. Membranes were detected using an enhanced chemiluminescence (ECL) system. Image-Pro Plus 6 was applied to quantify the density of immunoreactive bands.

### Immunohistochemistry and Immunofluorescence

Hearts were fixed with 4% paraformaldehyde, embedded in paraffin, and dissected into 5-μm-thick sections. Hematoxylin and eosin (H&E) staining was used to observe heart tissue morphology. MASSON staining was used to observe heart tissue fibrosis. Immunohistochemical staining was achieved by using the anti-collagen 1 antibody (1:100) (Abcam, cat# ab21286; RRID:AB_446161), anti-collagen 3 antibody (1:100) (Antibodies-Online, cat# ABIN285714; RRID:AB_10789249), anti-fibronectin antibody (1:100) (Abcam, cat# ab2413; RRID:AB_2262874), and anti-CD31 antibody (1:100) (Cell signaling technology, cat# 77699; RRID:AB_2722705) for 24 h at 4°C, and then HRP-conjugated anti-rabbit antibody for 1 h at room temperature. Then, the glass slides were incubated with 3, 3′-diaminobenzidine and counter-stained with hematoxylin. The following antibodies were used for co-localization immunohistochemistry analysis: anti-STAT3 (1:50) (Cell signaling technology, cat# 9139; RRID:AB_331757) co-labeled with anti–a-actinin (1:50) (Cell signaling technology, cat# 6487; RRID:AB_11179206). After incubation with Alexa 555– or Alexa 488–conjugated secondary antibodies (1:1000) (Thermo Fisher Scientific, cat# 21833; RRID:AB_2532155), all sections were observed with a laser confocal microscope (Zeiss LSM 710 system). Detection of the cell membrane was performed using fluorescein isothiocyanate–conjugated wheat germ agglutinin (Sigma-Aldrich).

### Plasma Biomarker

The blood samples of mice were collected and centrifuged at 2000rpm for 15 min. Then the serum were collected and stored frozen at −80°C in multiple aliquots until analysis. Biomarkers that reflect heart failure and a proinflammatory and profibrotic state, specifically brain natriuretic peptide (BNP), growth Stimulation expressed gene 2 (ST2) and interleukin 6 (IL-6) were chosen. The serum levels of BNP, ST2 and IL-6 were tested by mouse BNP enzyme-linked immunosorbent assay (ELISA) kit (Bio Tech Senxiong, catalog# Sxm117), mouse ST2 ELISA kit (Bio Tech Senxiong, catalog# Sxm106) and mouse IL-6 ELISA kit (Bio Tech Senxiong, catalog# Sxm032) following the manufacture's protocols.

### RNA-Sequence

The Cloud-seq biotechnology (Shanghai, China) helped to perform the RNA-sequencing. Data were analyzed by using R software on the Novelbrain platform (https://cloud.novelbrain.com). Under the guidance of Ensembl Gff gene annotation file, the HISAT2 method was used to compare the high-quality modified read with the reference genome (MiRbase v22). The DESeq2 method was used to calculate the fold change and P value based on the RNA count and RNAs expressed differently between the STAT3cKO mice hearts and WT mice hearts were finally placed. The standard between two groups was set as fold-change ≥2 or ≤-2 and *P*-value < 0.01.

### Statistical Analysis

All data were expressed as mean values ± standard error of the mean (SEM). Student's *t* test was performed to compare the difference between WT and STA3cKO groups, *P*-values < 0.05 were considered statistically significant.

## Results

### Cardiomyocyte-Specific Deletion of STAT3 Mice Were Generated by the Cre/FloxP Method

**Figure 1:**
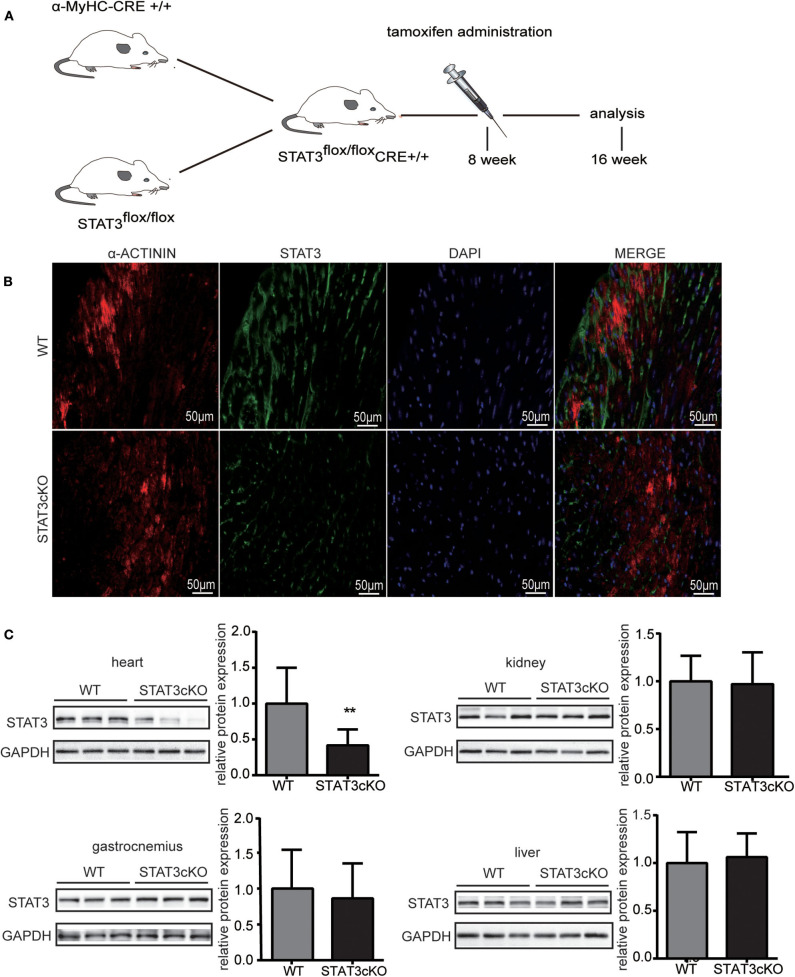
The stat3 gene was actually knock out in STATcKO mice. **(A)** Experimental design. STAT3^flox/flox^ Cre+ mice were administrated by tamoxifen at 8 weeks and analysis followed indicators at 16 weeks. **(B)** Representative mouse heart sections immunostained for α-ACTININ (red), STAT3 (green) and DAPI (blue) **(C)** Western blot analysis showed the levels of STAT3 in WT mice hearts, livers, kidneys and gastrocnemius (*n* = 3) and STAT3cKO mice hearts, livers, kidneys and gastrocnemius (*n* = 3). Compared with WT group, *P < 0.05 by Student's unpaired *t*-test. **P < 0.01 by Student's unpaired *t*-test.

The α-MyHC-Cre transgenic mice were crossed with mice having a loxP-flanked allele targeted STAT3 exons 3–4. The α-MyHC-Cre transgenic mice expressed Cre-recombinase in cardiomyocytes which was under the control of the α-myosin heavy chain (α-MyHC) gene promoter. Tamoxifen was administered for 8-wk-old flox/flox-Cre+ mice. All the indicators described below were tested when mice had grown to 4 months old (Figure 1A). Disruption of the STAT3 gene in cardiomyocytes was confirmed by immunofluorescence (Figure 1B) and western blot analysis (Figure 1C), and the STAT3 protein levels were significantly decreased in the heart of STAT3cKO mouse. In addition, we also demonstrated that there was no difference in the expression levels of STAT3 in the gastrocnemius, liver, and kidney of the STAT3cKO and WT mice (Figure 1C).

### Cardiomyocyte-Specific STAT3 Ablation Promoted Cardiac Fibrosis and Decreased Capillary Density

**Figure 2:**
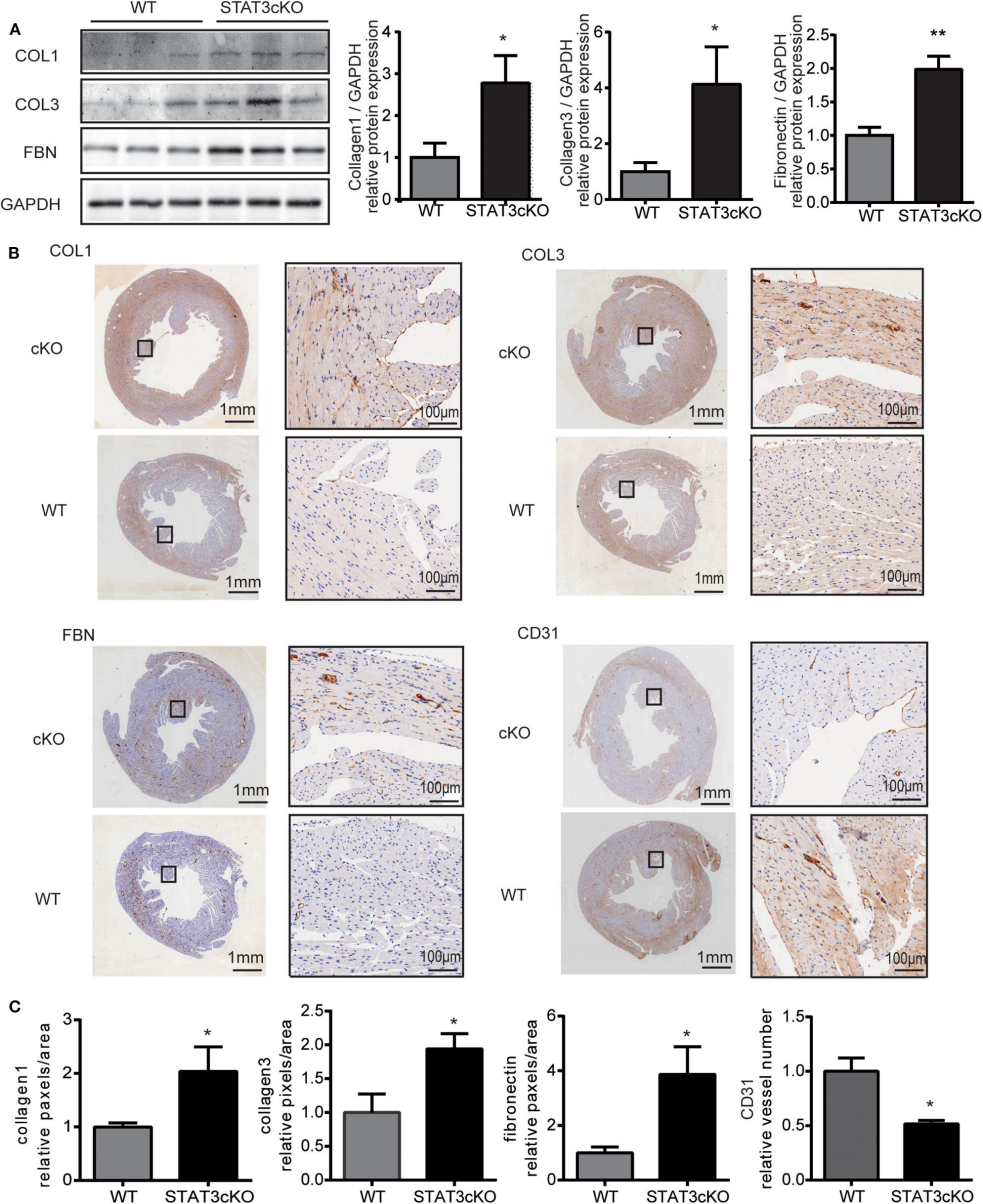
Cardiomyocyte-specific STAT3 ablation promotes cardiac fibrosis and reduces capillary density. **(A)** Western blot analysis showed the levels of collagen 1, collagen 3 and fibronectin in WT mice hearts (*n* = 3) and STAT3cKO mice hearts (*n* = 3). **(B)** Representative images of immunohistochemical staining of COL1, COL3, FBN, and CD31 in cardiac left ventricular tissue of WT mice and STAT3cKO mice. (Left panel of every image is original magnification. Right panel of every image is original magnification x10.) **(C)** Fixed quantity of immunohistochemical staining of COL1, COL3, FBN, and CD31 in cardiac left ventricular tissue of WT mice and STAT3cKO mice (*n* = 3). Compared with WT group, *P < 0.05 by Student's unpaired *t*-test. **P < 0.01 by Student's unpaired *t*-test.

Next, we tried to confirm whether STAT3cKO hearts had an altered response in cardiac fibrosis. Western blot analysis (Figure 2A) and histological analysis (Figures 2B,C and Supplementary Figure 1a) showed that cardiomyocyte-specific Stat3 ablation dramatically promoted cardiac fibrosis, as shown by collagen 1, collagen 3 and fibronectin. By histological and western blot analysis, we also found that the levels of CD31, a marker of capillary density were also significantly decreased in STAT3cKO mice (Figures 2B,C and Supplementary Figure 1b).

### Cardiomyocyte-Specific STAT3 Ablation Induced Cardiac Hypertrophy Without Affecting Blood Pressure

**Figure 3:**
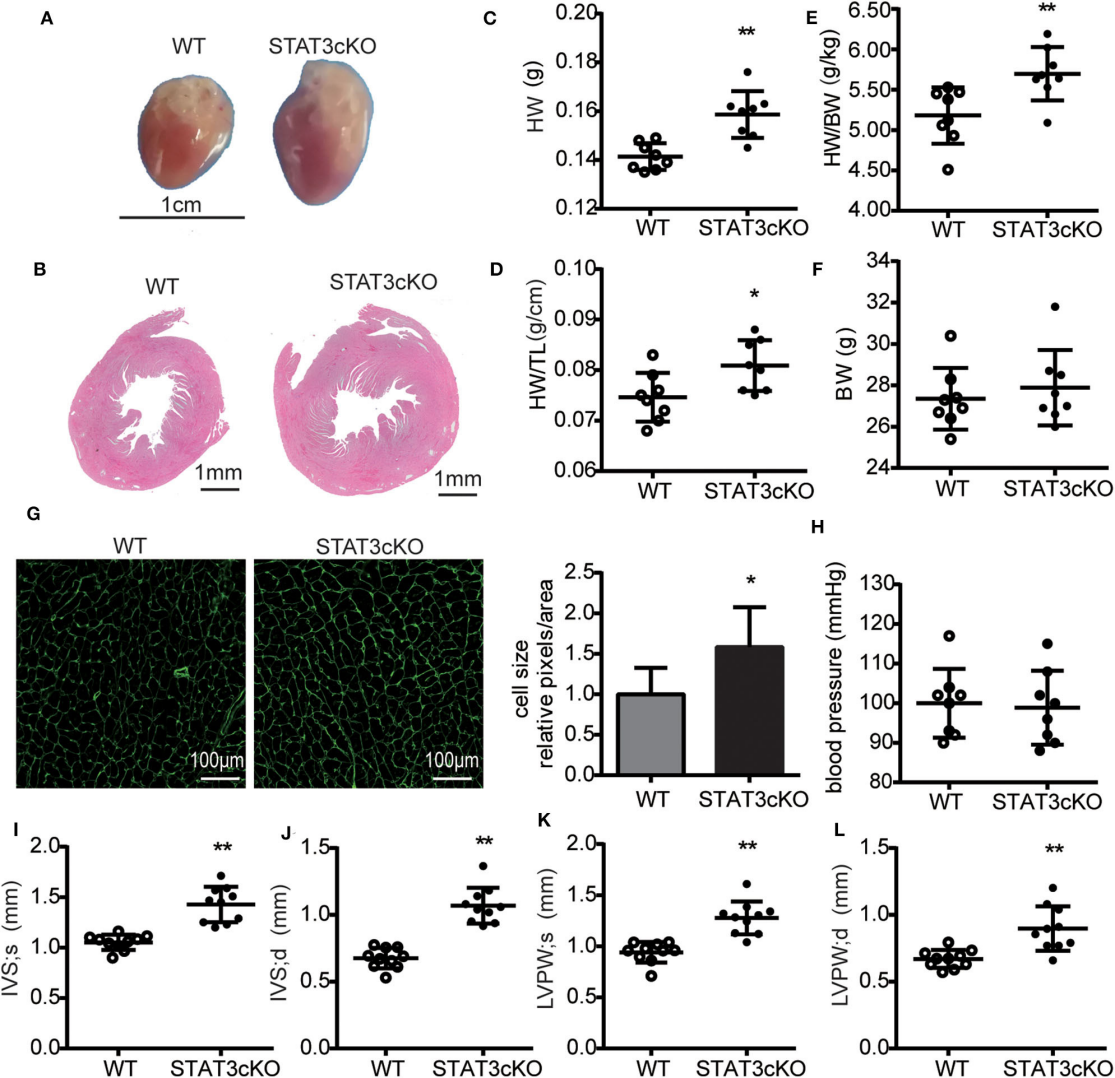
Cardiomyocyte-specific STAT3 ablation induces cardiac hypertrophy without affecting blood pressure. **(A)** Representative pictures of hearts from a WT mouse and STAT3cKO mouse. **(B)** Representative images of HE staining in cardiac left ventricular tissue of WT mice and STAT3cKO mice. **(C–F)** Heart weight (HW), ratio of heart weight to tibia length (HW/TL), ratio of heart weight to body weight (HW/BW), body weight in WT mice (*n* = 8) and STAT3cKO mice (*n* = 8). **(G)** Cardiomyocyte size as assessed by WGA staining in WT mice and STAT3cKO mice. **(H)** Blood pressure of WT mice (*n* = 8) and STAT3cKO mice (*n* = 8). **(I–L)** Levels of IVS;s, IVS;d, LVPW;s and LVPW;d in echocardiographic observations of WT mice (*n* = 10) and STAT3cKO mice (*n* = 10). Compared with WT group, *P < 0.05 by Student's unpaired *t*-test. **P < 0.01 by Student's unpaired *t*-test.

The STAT3cKO mice exhibit cardiac hypertrophy, as demonstrated by increased heart weight, heart weight to tibial length ratios and heart weight to body weight ratios (Figures 3C–E). These data were consistent with the observations seen in the photo shown in Figures 3A,B. Cardiac hypertrophic growth was accompanied by a larger cardiomyocyte size (Figure 3G). To assess cardiac hypertrophy further, 4-month-old STAT3cKO and WT mice were subjected to echocardiogram analysis. Compared with WT mice, STAT3cKO mice had increased interventricular septum thickness at end-systole (IVS; s) (Figure 3I) and end-diastole (IVS; d) (Figure 3J). Additionally, the left ventricular posterior wall thickness at end-systole (LVPW; s) and end-diastole (LVPW; d) significantly increased in STAT3cKO mice compared to WT mice (Figures 3K,L). Additionally, the blood pressures of all kinds of mice were similar (Figure 3H). Taken together, these findings suggested that cardiomyocyte-specific STAT3 ablation made mice developed cardiac hypertrophy without affecting blood pressure.

### Cardiomyocyte-Specific STAT3 Ablation Impaired Cardiac Diastolic Function

**Figure 4:**
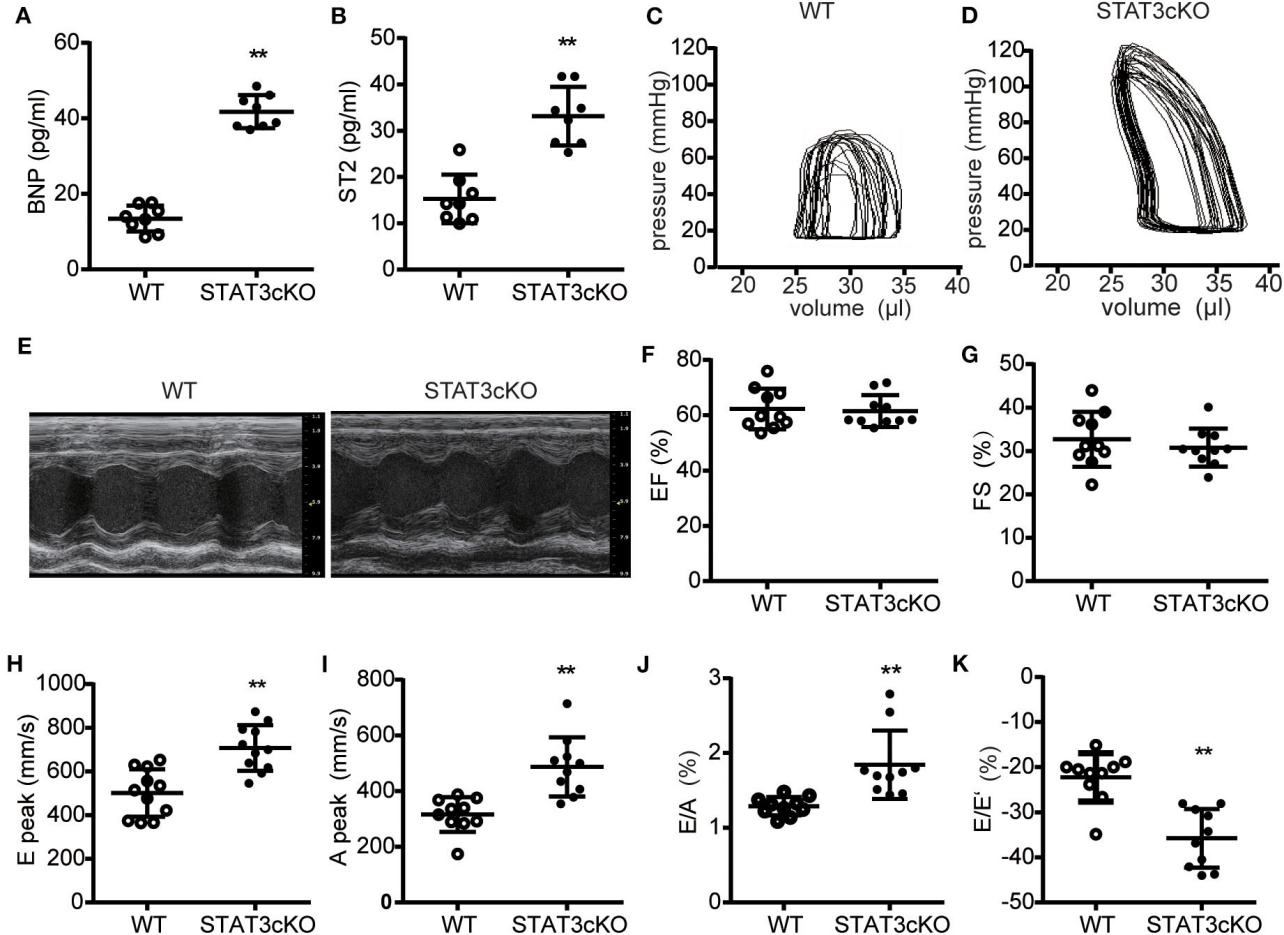
Cardiomyocyte-specific STAT3 ablation impaired cardiac diastolic function. **(A)** The levels of BNP concentration in WT mice (*n* = 8) and STAT3cKO mice (*n* = 8). **(B)** The levels of ST2 concentration in WT mice (*n* = 8) and STAT3cKO mice (*n* = 8). **(C,D)** Representative pictures of pressure-volume loop from a WT mouse and STAT3cKO mouse. **(E)** Representative left ventricular M-mode echocardiographic tracings of WT mice (*n* = 10) and STATcKO mice (*n* = 10). **(F,G)** Percentages of LVEF and FS in WT mice (*n* = 10) and STAT3cKO mice (*n* = 10). **(H–K)** Levels of E peak, A peak, E/A, E/E' in WT mice (*n* = 10) and STAT3cKO mice (*n* = 10). Compared with WT group, *P < 0.05 by Student's unpaired *t*-test. **P < 0.01 by Student's unpaired *t*-test.

We next tested some indicators of heart failure. The levels of serum brain natrium peptide (BNP), growth stimulation expression gene 2 (ST2) and interleukin 6 (IL-6) all increased heavily in STAT3cKO mice, which were the key biomarkers of heart failure (Figures 4A,B and Supplementary Figure 1f). Trans-mitral Doppler flow velocity showed higher trans-mitral valve velocity E peak (MV E) and A peak (MV A) in STAT3cKO mice than in WT mice, suggesting an increase in left ventricular (LV) chamber stiffness (Figures 4H,I). The ratio of mitral E velocity to mitral annular E' velocity (E/E'), a reliable predictor of LV end diastolic pressure, was elevated in STAT3cKO mice relative to WT mice (Figure 4K). The ratio of mitral E/A velocity was increased, also suggesting a restrictive LV filling pattern (Figure 4J), while the ejection fraction (EF) and fractional shortening were preserved (Figures 4E–G). These echo parameters were supported by a pressure volume analysis that revealed an increase in diastolic stiffness coefficient of the end diastolic pressure volume relation (EDPVR) in STAT3cKO mice (Figures 4C,D and Supplementary Figure 1e). In order to understand the condition of peripheral tissue edema, we tested the lung weight and HE staining of lung for all the mice. As a result, the HE staining showed intimal thickening of pulmonary capillaries in STAT3cKO mice and lung weight of STAT3cKO mice was heavier than that of WT mice (Supplementary Figures 1c,d). These data suggested that STAT3cKO mice have pulmonary edema to a certain extent. In summary, the STAT3cKO mice developed a deep degree of diastolic dysfunction while systolic function was preserved.

### Cardiomyocyte-Specific Deletion of STAT3 Reduced Myocardial PKG Levels and Eventually Impaired Cardiac Diastolic Function

**Figure 5:**
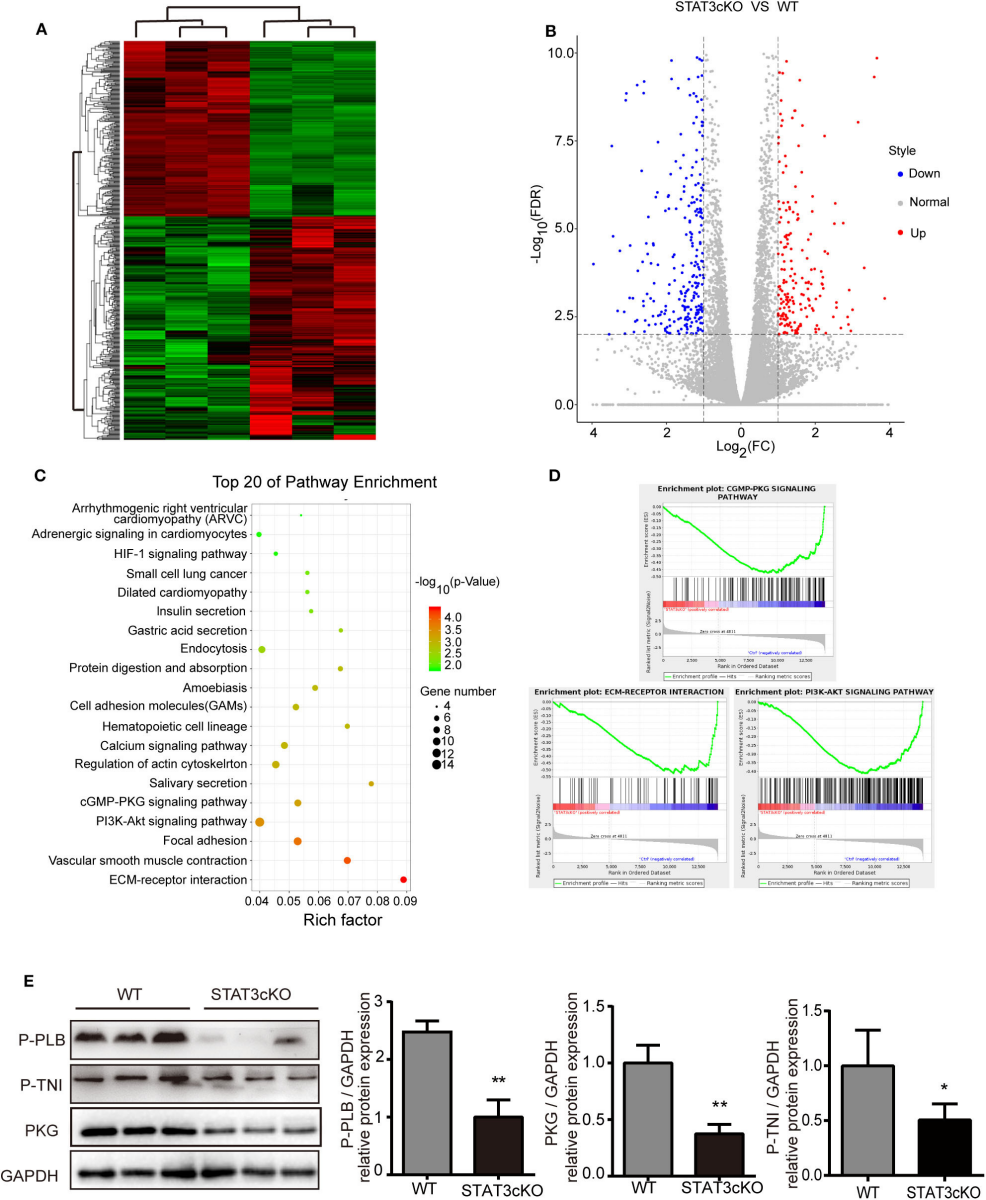
Cardiomyocyte-specific deletion of Stat3 reduced myocardial PKG levels and RNA-sequencing of STAT3cKO mice hearts suggested potential pathogenesis of HFpEF. **(A)** The hierarchical clustering method was used to identify the consistency of dysregulated mRNAs in STAT3cKO (*n* = 3) and WT (*n* = 3) mice hearts. **(B)** The colcano map showed the 223 up-regulated gens and 285 down-regulated gens in STAT3cKO and WT mice hearts (Fold change≥2 and *P* < 0.01). **(C)** The top 20 of pathway enrichment were ananlyzed by Kyoto Encyclopedia of Genes and Genomes analysis. **(D)** The enrichment plot of cGMP-PKG signaling pathway, ECM-RECEPTOR interaction, and PI3K-AKT signaling pathway enrichment were ananlyzed by Gene Set Enrichment Analysis. **(E)** Western blot analysis showed the levels of PKG, P-PLB and P-TNI in WT mice hearts (*n* = 3) and STAT3cKO mice hearts (*n* = 3). Compared with WT group, *P < 0.05 by Student's unpaired *t*-test. **P < 0.01 by Student's unpaired *t*-test.

Based on the data above, we tried to explore the possible mechanisms of cardiac diastolic dysfunction in STAT3cKO mice. RNA sequence of the heart tissue of STAT3cKO mice was tested. Bioinformatic analysis [including Gene Ontology (GO) analysis, Kyoto Encyclopedia of Genes and Genomes (KEGG) analysis and Gene Set Enrichment Analysis (GSEA)] were performed to identify potential effects of differential genes in STAT3cKO mice hearts. The hierarchical clustering method was used to confirm the consistency of dysregulated mRNAs in STAT3cKO mice (*N* = 3) and WT mice (*N* = 3) hearts (Figure 5A). A total of 508 dysregulated genes (including 223 up-regulated genes and 285 down-regulated genes) were distinguished via the expression analysis, (Figure 5B). KEGG analysis were done to identify the relevant pathways for predicting target mRNAs (Figure 5C). The top five associated pathways were ECM-receptor interaction, Vascular smooth muscle contraction, Focal adhesion, PI3K-Akt signaling pathway and cGMP-PKG signaling pathway. Studies showed that cyclic guanosine monophosphate (cGMP)-protein kinase G (PKG) signaling pathway has been provided novel perspectives on HFpEF (13, 19). ECM-receptor interaction and PI3K-Akt signaling pathway may influnce cardiac function (1). So GSEA analysis was applied to further explore the hub pathway. The GSEA analysis showed that cGMP-PKG signaling pathway had a better enrichment score and gene original size comparing with ECM-receptor interaction and PI3K-Akt signaling pathway (Figure 5D). So we focused on cGMP-PKG signaling pathway. Through KEGG map of cGMP-PKG signaling pathway, we found that there were 15 downregulated genes (including PKG, βAR, ATPase and so on) and two upregulated genes (ROS and βMHC) in STAT3cKO mice hearts (Supplementary Figure 2). As a result, the 17 differential gens in cGMP-PKG signaling pathway affected cardiac systolic function by increasing cardiac hyprertrophy, increasing intracellular free calcium, decreasing cardioprotection of mitochondria, inducing endothelial dysfunction and so on.

Consistent with the results of RNA sequence, the protein levels of PKG were significantly down-regulated in STAT3cKO hearts. Additionally, the levels of phosphorylation of troponin I [p(S23/24)-TNI] and phospholamban [p(S16)-PLB] which were two of the major events underlying β-adrenergic–mediated signaling (17, 22), also decreased obviously in STAT3cKO mice hearts (Figure 5E). These results indicated that loss of STAT3 in cardiomyocyte ultimately promoted cardiac diastolic dysfunction partly due to the reduced myocardial PKG levels.

## Discussion

HFpEF is a fetal disease and there is not enough effective clinical therapies (23). Considering the limitations of present animal models, we developed a murine model of cardiomyocyte-specific deletion of STAT3 that recapitulated the clinical characteristics of HFpEF.

The disruptions which we performed to duplicate the clinical characterization are on the basis of pathophysiological observations of human condition. Changes in cardiac passive stiffness have been confirmed in HFpEF patients, and our murine model takes full advantage of this feature to manage increased cardiac passive stiffness from the start. By contrast, the traditional HFpEF animal models imitate specific characteristics matching particular HFpEF populations such as hypertension, obesity, diabetes mellitus, and aging. For example, a typical hypertension animal model imitated HFpEF-the Dahl salt-sensitive rat (8). Actually, this kind of model can implicate the renin–angiotensin–aldosterone system in HFpEF. However, with increasing HFpEF research, therapies targeted RAAS system have little clinical value for HFpEF patients (23, 24). In addition to this, murine diabetes mellitus and obesity models such as the Akita mouse (Ins2 Akitaþ/-) (9, 10), glucosamine-nitrosourea streptozotocin (STZ) mice (9, 10), ob/ob (11), and db/db (25) mice all exhibited some phenotypes of HFpEF (26–29). In fact, these are more suitable as metabolic syndrome animal models and give little help for research on preclinical evaluation of potentially novel therapeutic strategies.

In the present study, we demonstrated that animals lacking cardiomyocyte expression of STAT3 were more likely to develop HFpEF (Figure 4). More significantly, we found that cardiac deletion of STAT3 led to cardiac passive stiffness. More and more studies have shown that cardiac diastolic function can be affected by LV stiffness, which includes ECM-based and titin-based passive stiffness (30, 31). Our study focused on the former. Cardiomyocyte-specific deletion of STAT3 dramatically increased the levels of the collagen 1 and collagen 3 which were the major ECM components contributing to cardiac passive stiffness (Figure 2) These results were completely in line with the characteristic of increased ECM-based cardiac stiffness in HFpEF (19, 20). Additionally, previous studies showed that the release of catecholamines activated the PKA pathway through β-adrenergic receptors (β ARs) and induces the phosphorylation of the spring-like domains of titin. Subsequently, increased flexibility and compliance of titin extends the physiological length of the sarcomere and improves the diastolic function of the heart (12, 13, 21, 32–34). We found cardiomyocyte-specific deletion of STAT3 down regulated β-adrenergic–mediated signaling (Figure 5). However, the phosphorylational state of titin remains unclear in STAT3cKO mice, which need to be further investigated.

Reporters suggested that HFpEF patients showed reduced myocardial PKG levels and lower cGMP concentration comparing with HFrEF patients (13). As the main protein kinases, PKG phosphorylates a great deal of proteins, showing a variety of downstream effects such as enhancing intracellular diastolic calcium reuptake by phosphorylation of phospholamban, inhibiting hypertrophic signaling via inhibition of G-protein coupled receptors, and stimulation of left ventricular relaxation and distensibility by phosphorylation of troponin I (TnI) and titin, and so on (35). These alterations were clearly consistent with our results (Figure 5 and Supplementary Figure 2). Alterations in cardiomyocyte cGMP-PKG pathway ultimately increased interstitial fibrosis, cardiomyocyte hypertrophy and finally weaken cardiac diastolic function through associated downstream effects explaining above.

Early studies showed that global ablation of the STAT3 gene in mice results in embryonic lethality during embryonic development (36). Other studies have shown that cardiomyocyte-specific deletion of mouse STAT3 gene did not affect the heart structure and function of young mice (16). So we generated cardiomyocyte-specific STAT3 knock-out models in 8-wk-old mice and tested all the data when the mice were 16–wk old. In addition to this, as a temporary cardiomyopathy caused by Cre expression existed about 4 weeks, all mice should be permitted to recover for 6 weeks after the last tamoxifen injection (37). In our study, tamoxifen had no effect on the 16-wk old STAT3cKO mice. Although the STAT3cKO mice model imitated one of the most common comorbidity in the human setting, there still exerts some limitations in the model. The main limitation is that as the main member of signal transducer and activator of transcription family, STAT3 regulates a large number of biological functions primarily in response to extracellular signaling molecules such as cytokines and growth factors (Figure 5). As STAT3 is the key cellular molecule, it is not certain whether other impaired signaling pathways would affect cardiac diastolic function in a STAT3cKO mouse model. Another important limitation is that we did not exam the advanced performance in STAT3cKO mice. In our study, the cardiac systolic function remained normal in 16-wk-old STAT3cKO mice. Previous studies suggested that adult Stat3cKO mice spontaneously developed heavily myocardial fibrosis and eventually HFrEF at 36 weeks (16). Actually, the transformation of HF phenotype in STAT3cKO mice with aging is consistent with clinical HF patients. Under the physiological stresses or other factors, HFpEF patients may develop to heart failiure with reduced eject fraction.

In summary, we have shown that cardiomyocyte-specific deletion of STAT3 caused cardiac fibrosis, and hypertrophy. As a result, in mice with myocardial-specific deletion of STAT3, cardiac diastolic functions were impaired, while systolic function remained normal. Moreover, we have revealed that STAT3 regulates the levels of PKG, that affects the cardiac ECM-based passive stiffness. Together, these data clearly have demonstrated that mice with cardiomyocyte-specific deletion of STAT3 are a successful HFpEF animal model, which will contribute to the development of HFpEF research on treatment in the future.

## Data Availability Statement

The datasets generated for this study can be found in online repositories. The names of the repository/repositories and accession number(s) can be found in the article/Supplementary Material.

## Ethics Statement

The animal study was reviewed and approved by the Committee on the Ethics of Animal Experiments of the Shanghai Jiao Tong University School of Medicine. Written informed consent was obtained from the owners for the participation of their animals in this study.

## Author Contributions

WZ, YC, KY, and WJ were responsible for the design of the study and the writing the manuscript. WY and YH were responsible for data analysiswork. ZW, FH, and ZQ were responsible for the edit of the manuscript. All authors read and approved the final manuscript.

## Conflict of Interest

The authors declare that the research was conducted in the absence of any commercial or financial relationships that could be construed as a potential conflict of interest.

## Supplementary Material

Supplementary Figure 1 Cardiomyocyte-specific STAT3 ablation impaired cardiac diastolic function. **(a)** Representative images of MASSON staining in heart of WT mice and STAT3cKO mice. **(b)** Western blot analysis showed the levels of CD31 in WT mice hearts (*n* = 3) and STAT3cKO mice hearts (*n* = 3) **(c)** Representative images of HE staining in lung of WT mice and STAT3cKO mice. **(d)** Lung weight of WT mice (*n* = 8) and STAT3cKO mice (*n* = 8). **(e)** The diastolic stiffness coefficient of the end diastolic pressure volume relation (EDPVR) by pressure-volum analysis in STAT3cKO mice (*n* = 7) and WT mice (*n* = 7). **(f)** The levels of IL-6 concentration in WT mice (*n* = 8) and STAT3cKO mice (*n* = 8). ^*^*P* < 0.05 by Student's unpaired *t*-test. ^**^*P* < 0.01 by Student's unpaired *t*-test.

Supplementary Figure 2 The cGMP-PKG signaling pathway might be involved in the pathogenesis of HFpEF via regulating different biological functions. The cGMP-PKG signaling pathway KEGG map showed the 15 downregulated genes and two upregulated genes.
