# Supplementary figures and images for: Effects of Cardiomyocyte-Specific Deletion of STAT3–A Murine Model of Heart Failure With Preserved Ejection Fraction
Source: Front Cardiovasc Med. 2020 Dec 7;7:613123. doi: 10.3389/fcvm.2020.613123 (PMC7750364; doi:10.3389/fcvm.2020.613123)

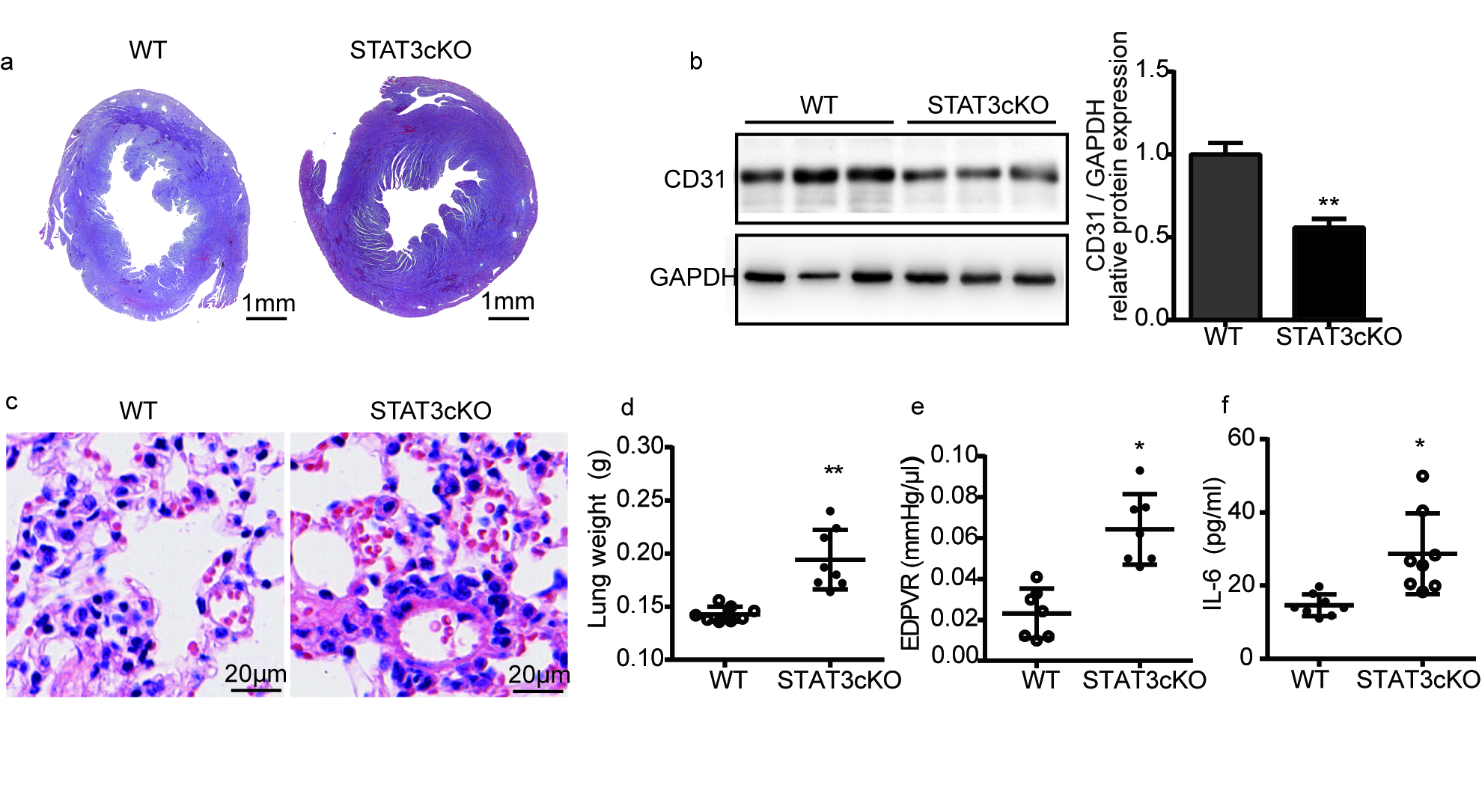

Supplement: Supplementary Figure 1 — Cardiomyocyte-specific STAT3 ablation impaired cardiac diastolic function. (a) Representative images of MASSON staining in heart of WT mice and STAT3cKO mice. (b) Western blot analysis showed the levels of CD31 in WT mice hearts (n = 3) and STAT3cKO mice hearts (n = 3) (c) Representative images of HE staining in lung of WT mice and STAT3cKO mice. (d) Lung weight of WT mice (n = 8) and STAT3cKO mice (n = 8). (e) The diastolic stiffness coefficient of the end diastolic pressure volume relation (EDPVR) by pressure-volum analysis in STAT3cKO mice (n = 7) and WT mice (n = 7). (f) The levels of IL-6 concentration in WT mice (n = 8) and STAT3cKO mice (n = 8). *P < 0.05 by Student's unpaired t-test. **P < 0.01 by Student's unpaired t-test. [file Image_1.TIF]

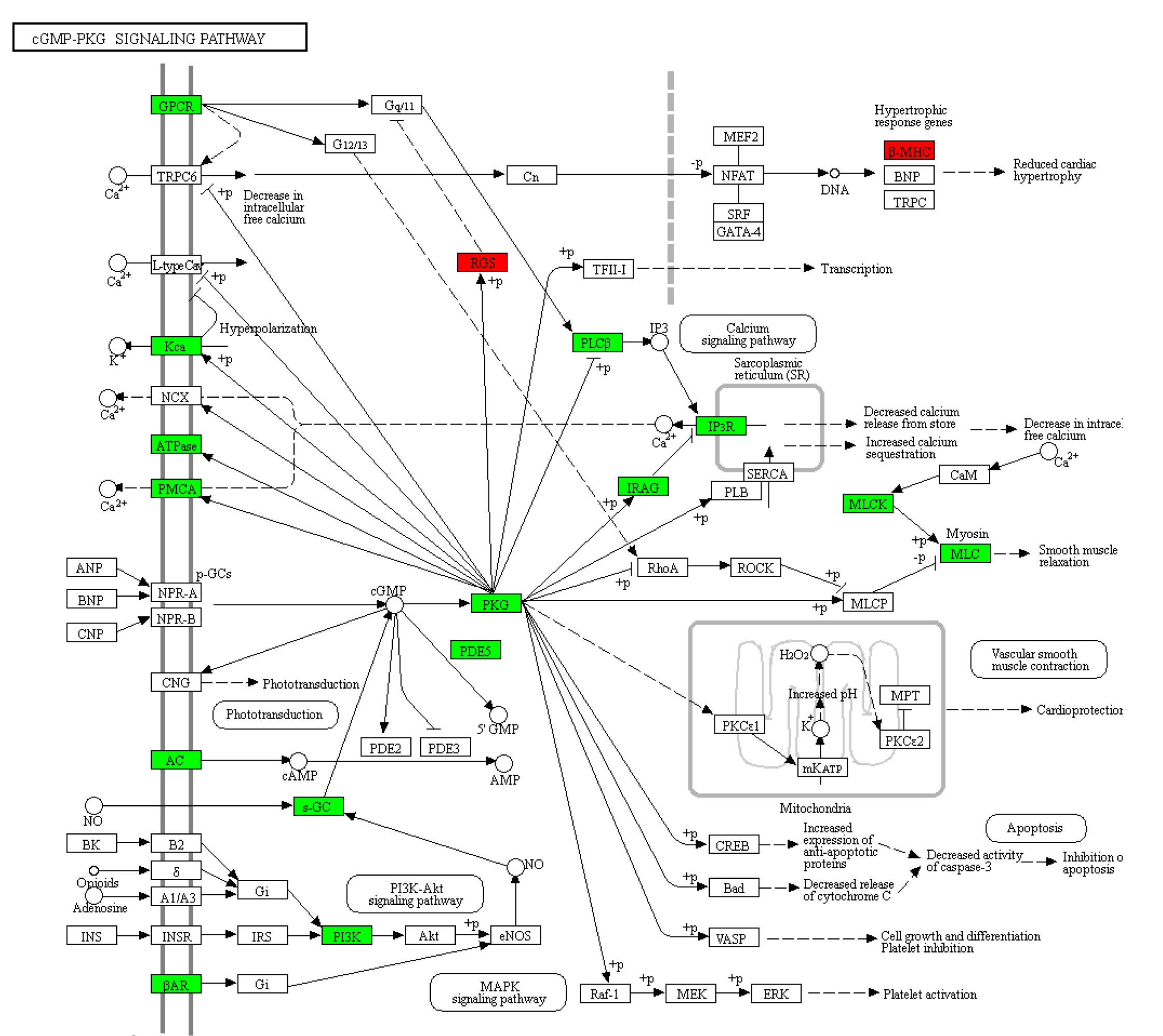

Supplement: Supplementary Figure 2 — The cGMP-PKG signaling pathway might be involved in the pathogenesis of HFpEF via regulating different biological functions. The cGMP-PKG signaling pathway KEGG map showed the 15 downregulated genes and two upregulated genes. [file Image_2.TIF]
